# Understanding Phantom Shocks in Implantable Cardioverter-Defibrillator Recipients

**DOI:** 10.7759/cureus.53161

**Published:** 2024-01-29

**Authors:** Jason Galo, Rafey Feroze, Talal Almas, Julianna Morera, Jayakumar Sahadevan

**Affiliations:** 1 Cardiology, University Hospitals Cleveland Medical Center, Case Western Reserve University, Cleveland, USA; 2 Internal Medicine, University Hospitals Cleveland Medical Center, Case Western Reserve University, Cleveland, USA; 3 Arts and Sciences, University of Miami, Coral Gables, USA; 4 Electrophysiology, Case Western Reserve University, Cleveland, USA

**Keywords:** therapy shock differentiation, cardiac-psychological nexus, psychological distress, implantable cardioverter-defibrillator (icd), phantom shocks

## Abstract

Phantom shocks in implantable cardioverter-defibrillator (ICD) recipients create a complex nexus between cardiac treatment and psychological distress. These sensations, mimicking therapeutic shocks without device activation, deeply affect patients' functionality and well-being. Heightened anxiety, depression, and hopelessness predispose individuals to these occurrences, posing significant challenges. This article delves into the intricate nature of phantom shocks, highlighting subtle clinical cues to differentiate them from genuine therapy shocks. Through a case study of a 75-year-old male with recurrent ICD shocks, diagnosed eventually with phantom shocks, the interplay between psychological distress and physical sensations is underscored. Urgent intervention to address the patient's anxiety and depression using psychotherapy and antidepressants became imperative. The case underscores the immense psychological toll of phantom shocks, exacerbating fear, hopelessness, and post-traumatic stress disorder (PTSD). Despite treatment attempts, their impact persisted, leading to a shift to comfort-focused care. While research identifies factors such as education levels and prior therapy, predicting and managing phantom shocks remains challenging. This article stresses the need for clinician vigilance, urging proactive identification and tailored interventions to mitigate the profound effects of phantom shocks. The current research landscape lacks comprehensive strategies, necessitating further exploration and targeted therapies to restore patient well-being. In conclusion, comprehensive understanding and specialized care for phantom shocks in ICD recipients, addressing both cardiac and psychological aspects, are imperative. Early recognition and tailored interventions offer promise in alleviating their adverse effects, reinstating patient control, and improving their quality of life.

## Introduction

The implantable cardioverter-defibrillator (ICD) stands as a sophisticated and miniaturized device with the remarkable ability to automatically sense and terminate life-threatening ventricular arrhythmias. This cutting-edge technology plays a pivotal role in preventing arrhythmic sudden cardiac death (SCD) and has proven to be a lifesaving intervention for thousands of patients globally [1]. Phantom shocks represent a perplexing and underexplored occurrence among patients with ICDs [2,3]. These sensations mimic the experience of receiving a shock from the device without an actual therapeutic intervention [4]. Distinguishing phantom shocks from genuine therapy shocks relies on nuanced clinical markers, emphasizing the need for deeper comprehension and refined diagnostic approaches in managing these incidents [5]. This article elucidates the intricate nature of phantom shocks, exploring their clinical characteristics, potential underlying mechanisms, diagnostic challenges, and management strategies within the realm of cardiac electrophysiology.

## Case presentation

Phantom shocks manifest as perceived electrical jolts or sensations resembling authentic therapy shocks in ICD recipients [4]. Distinguishing between the two necessitates a nuanced assessment, considering subjective patient experiences, device interrogation, and electrocardiographic correlation. Subtle clinical nuances, including the absence of arrhythmia detection, lack of device logs, and absence of associated hemodynamic changes, serve as pivotal discriminators in identifying phantom shocks [5]. A 75-year-old male, burdened by a history of severe ischemic cardiomyopathy, coronary artery disease managed through coronary artery bypass grafts (CABG) and percutaneous coronary intervention, and recurrent ventricular tachycardia (VT) storm necessitating ICD placement for secondary prevention, presented to the emergency department for the second time in two months, reporting an ICD shock.

The patient's psychiatric history revealed a notable diagnosis of adjustment disorder with mixed depression and anxiety, potentially leaning toward major depression. He exhibited symptoms such as sadness, irritability, and suicidal ideation without concrete intention or plan, coupled with poor appetite, disrupted sleep patterns, and self-blame. The psychiatric evaluation was intricate due to the presence of Cluster B personality traits indicative of borderline personality traits. These traits encompass frantic attempts to avoid abandonment, identity disturbances, emotional instability, persistent feelings of emptiness, expressions of anger, and stress-related paranoid ideation.

The patient's surgical history encompassed a CABG coupled with concurrent mitral valve replacement and an abdominal aortic aneurysm (AAA) status post endovascular aneurysm repair (EVAR). In terms of family medical background, there was a history of myocardial infarction (MI) in the father at the age of 70 and type 2 diabetes in the mother. The patient's medication regimen consisted of amiodarone 200 mg daily, carvedilol 3.125 mg twice daily, clopidogrel 75 mg daily, ranolazine 500 mg twice daily, rivaroxaban 15 mg daily, rosuvastatin 20 mg daily, sacubitril/valsartan 24 mg/26mg twice daily, and sertraline 25 mg daily.

Socially, the patient had a relevant history of smoking, amounting to 10 pack-years with cessation 30 years prior to admission. He reported minimal alcohol consumption, with one glass of wine every two to three weeks. Living alone at home, the patient's occupation was a security guard. Notably, he recently ended a 17-year relationship with a long-term girlfriend. While he had two daughters as part of his support system, both resided a few hours away.

Contrary to previous episodes, characterized by prodromal symptoms of lightheadedness and dizziness, the recent shocks were abrupt and unanticipated, described by the patient as a forceful sensation akin to "a strong fist pump" in his chest. Device interrogation yielded no evidence of VT or ventricular fibrillation (VF) and no history of treated episodes.

**Figure 1:**
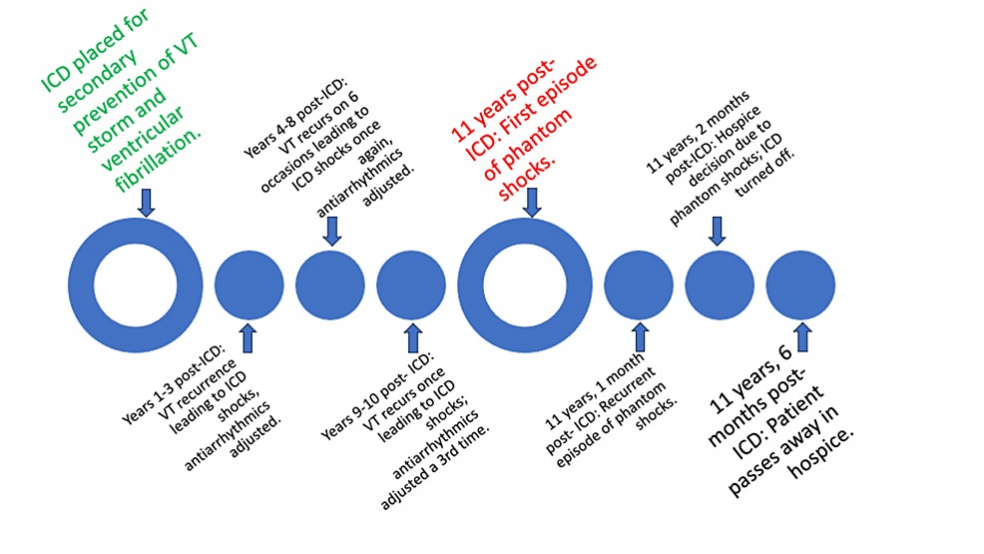
Case timeline ICD=Implantable Cardioverter-Defibrillator; VT=Ventricular Tachycardia

During hospitalization, the patient conveyed feelings of burnout, depression, and overwhelming anxiety stemming from the challenges of adapting to life with an ICD. A comprehensive assessment led to a diagnosis of phantom shock, highlighting the interplay between his psychological distress and somatic experiences. Addressing the patient's underlying anxiety, depression, and psychological turmoil became paramount. Initiating a treatment plan encompassing psychotherapy and antidepressants aimed to alleviate his emotional burden and enhance coping strategies. Considering the advanced nature of his heart failure and guarded prognosis, a sensitive goals-of-care discussion ensued. In line with the patient's wishes and in pursuit of comfort-focused care, he elected to deactivate his ICD, transitioning to hospice care for personalized end-of-life support (Figure 1).

## Discussion

The phenomenon of phantom shocks, where patients perceive the sensation of receiving ICD therapy in the absence of documented device activation, presents a multifaceted challenge in clinical practice [6]. Various factors, including higher education levels and prior experience with therapy shocks, have been associated with the development of these enigmatic sensations (Table 1) [5].

**Table 1:** Exploring factors linked to the onset of phantom shocks Table Credits: Jason Galo, MD (Author)

Exploring factors linked to the onset of phantom shocks
Higher education level greater than bachelor’s degree (OR: 2.8 [95% CI: 1.2–6.2]; p = 0.03)
Higher anxiety in the HADS scores (15 ± 5.7 versus 8.8 ± 7.4 points; p = < 0.0001)
Subjective patient impression that the information provided before device placement was not sufficient (22.2% versus 5.0%; p = 0.004)
Need for psychological support after ICD implantation (25.9% versus 3.3%; p = < 0.0001)
More likely to have considered switching off their ICD in near end-of-life situations (59.3% versus 29.5%; p = < 0.002)
History of adequate ICD shocks (OR: 59.0 [95% CI: 17–205]; p =< 0.0001)
HADS scores=Hospital Anxiety and Depression Scale; ICD=Implantable Cardioverter-Defibrillator; PS=Phantom Shocks. Source: [5]

**Figure 2:**
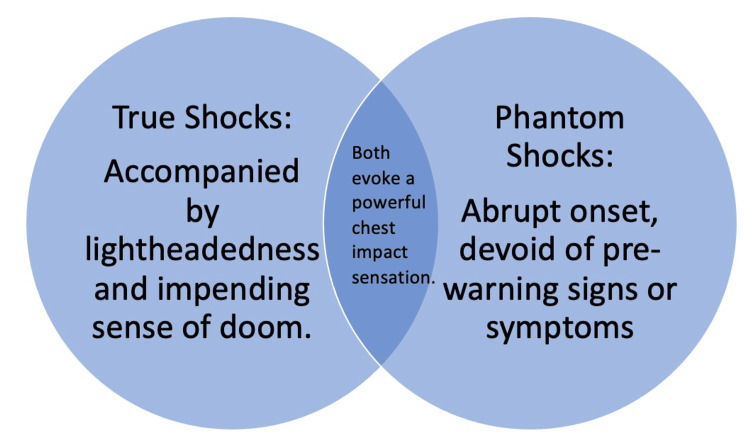
Distinguishing genuine ICD shocks from phantom shocks Image Credits: Jason Galo, MD (Author)

Patients undergoing ICD implantation navigate a spectrum of psychological, physical, and social adjustments, often grappling with heightened levels of anxiety, depression, and a sense of hopelessness [6,7]. In this case, we present an individual with a pre-existing anxiety and potentially depressive disorder, who had previously experienced genuine therapy shocks and ultimately developed a profound fear of anticipated shocks. A comprehensive psychiatric evaluation was undertaken, leading to the conclusion that the phantom shocks were indicative of or contributing to ICD maladjustment. Although both the actual and phantom shocks elicited similar sensations, the former was consistently preceded by lightheadedness and an impending sense of doom, indicative of symptomatic VT or VF. In stark contrast, the phantom shocks lacked any associated arrhythmias or premonitory symptoms (Figure 2).

These episodes exacerbated the patient's fear, hopelessness, and debilitating fatigue, culminating in evidence of post-traumatic stress disorder (PTSD). The psychological impact of phantom shocks cannot be understated, often leading to clinically significant depression, anxiety, and even agoraphobia rooted in the fear of prospective shocks [6]. This mirrors the symptoms observed in certain patients who have experienced actual ICD shocks, encompassing agoraphobia, cognitive distortions, and heightened vigilance to physical sensations [7]. Attempts to address the underlying anxiety through psycho- and pharmacotherapy yielded limited results. This perplexing phenomenon not only challenges patients but also confounds clinicians, necessitating further exploration into its pathophysiology, predisposing factors, and optimal treatment approaches [8]. Research directions in the field of phantom shocks associated with ICDs encompass a multidimensional approach. Investigations could delve into the psychosocial impact of phantom shocks, exploring correlations with mental health outcomes. Neurological factors, such as understanding the neural mechanisms behind these sensations, present an avenue for collaboration with neuroscientists. Additionally, researchers might scrutinize the influence of device programming, patient-specific factors, and potential interventions for managing and reducing phantom shocks. Long-term outcomes, patient education, and comparative studies across ICD models could shed light on effective strategies for mitigating the negative effects of these events. The role of remote monitoring and adopting multidisciplinary approaches involving cardiologists, psychologists, and neurologists further adds to the comprehensive exploration of this phenomenon.

In a study by Kraaier et al., involving 629 ICD recipients, 32 reported phantom shock sensations without evidence of therapy shocks upon device interrogation. However, no definitive predictors for phantom shocks were identified, and none of the patients experienced debilitation severe enough to choose comfort-focused care, as observed in this case [4]. Additionally, a case report involving a patient with a wearable cardioverter defibrillator suggests that the permanence of an ICD might not solely trigger these phantom sensations and subsequent anxiety [2]. Notably, the successful management of nocturnal phantom shock with zolpidem in another case report underscores potential treatment avenues for similar cases [9]. Finally, another case report describes a patient similar to ours, diagnosed with adjustment disorder with mixed anxiety and depressed mood after experiencing phantom shocks [10]. The treatment approach in that case focused on addressing the combined symptomatology (insomnia, anxiety, and depression) and involved initiating mirtazapine, selected for its effectiveness in treating depression alongside coexisting anxiety and insomnia [10]. While considering mirtazapine as a potential option for our patient, its suitability was ruled out due to the rotational nocturnal nature of his job as a security guard.

To our knowledge, this case represents an unprecedented severity of debilitation induced by phantom shocks, culminating in recurrent admissions and a transition to comfort-focused care due to overwhelming psychosocial stress. It underscores the critical need for vigilant monitoring of ICD recipients for signs of depression, anxiety, and PTSD during routine clinical visits, necessitating prompt referrals to experienced mental health professionals when warranted. Furthermore, research remains scarce regarding the efficacy of pharmacotherapy or psychotherapy in managing this distressing condition, emphasizing the need for comprehensive investigation into potential treatment modalities.

## Conclusions

Phantom shocks, a puzzling occurrence in those with ICDs, blend cardiac treatment with psychological distress. These sensations, mimicking therapeutic shocks without device activation, significantly hinder patients' daily function and quality of life. Higher levels of anxiety, depression, and hopelessness often predispose ICD patients to these shocks, emphasizing the complex interplay between cardiac and psychological health. Clinicians must recognize and understand phantom shocks to screen, diagnose, and treat affected patients effectively. Recognizing and addressing phantom shocks are crucial in comprehensive care for ICD recipients. In navigating the intricate intersection of cardiac and psychological well-being, a nuanced understanding of phantom shocks becomes imperative for clinicians. By acknowledging and addressing these enigmatic occurrences, healthcare providers can enhance the overall care and quality of life for individuals with ICDs.
